# Development of the Sensing Platform for Protein Tyrosine Kinase Activity

**DOI:** 10.3390/bios11070240

**Published:** 2021-07-15

**Authors:** Lan-Yi Wei, Wei Lin, Bey-Fen Leo, Lik-Voon Kiew, Chia-Ching Chang, Chiun-Jye Yuan

**Affiliations:** 1 Department of Biological Science and Technology, National Yang Ming Chiao Tung University, Hsinchu 30068, Taiwan; lanyi.bce99g@nctu.edu.tw (L.-Y.W.); squall5838.bt07g@nctu.edu.tw (W.L.); lvkiew@um.edu.my (L.-V.K.); ccchang01@nctu.edu.tw (C.-C.C.); 2 Faculty of Medicine, University of Malaya, Kuala Lumpur 50603, Malaysia; beyfenleo@um.edu.my; 3 Taiwan-Malaysia Semiconductor and Biomedical Oversea Science and Technology Innovation Center, National Yang Ming Chiao Tung University, Hsinchu 30068, Taiwan; 4 Department of Pharmacology, Faculty of Medicine, University of Malaya, Kuala Lumpur 50603, Malaysia; 5 Center for Intelligent Drug Systems and Smart Bio-devices (IDS2B), National Yang Ming Chiao Tung University, Hsinchu 30068, Taiwan; 6 Department of Electrophysics, National Yang Ming Chiao Tung University, Hsinchu 30010, Taiwan; 7 Institute of Physics, Academia Sinica, Nankang, Taipei 11529, Taiwan; 8 Institute of Molecular Medicine and Bioengineering, National Yang Ming Chiao Tung University, Hsinchu 30068, Taiwan

**Keywords:** biosensor, electrochemical analysis, tyrosine kinase

## Abstract

A miniature tyrosinase-based electrochemical sensing platform for label-free detection of protein tyrosine kinase activity was developed in this study. The developed miniature sensing platform can detect the substrate peptides for tyrosine kinases, such as c-Src, Hck and Her2, in a low sample volume (1–2 μL). The developed sensing platform exhibited a high reproducibility for repetitive measurement with an RSD (relative standard deviation) of 6.6%. The developed sensing platform can detect the Hck and Her2 in a linear range of 1–200 U/mL with the detection limit of 1 U/mL. The sensing platform was also effective in assessing the specificity and efficacies of the inhibitors for protein tyrosine kinases. This is demonstrated by the detection of significant inhibition of Hck (~88.1%, but not Her2) by the Src inhibitor 1, an inhibitor for Src family kinases, as well as the significant inhibition of Her2 (~91%, but not Hck) by CP-724714 through the platform. These results suggest the potential of the developed miniature sensing platform as an effective tool for detecting different protein tyrosine kinase activity and for accessing the inhibitory effect of various inhibitors to these kinases.

## 1. Introduction

Protein tyrosine kinases are one of the phosphotransferase families that transfer γ-phosphate of adenosine triphosphate (ATP) to the tyrosine residues of the target proteins [1]. Tyrosine phosphorylation has been shown to play essential roles in many cellular events, such as cell proliferation and differentiation, protein synthesis, cell cycle, embryo development, cell migration and apoptosis [2,3,4,5]. Dysregulation of protein tyrosine kinases, either by overexpression or overactivation, leads to many diseases, such as diabetes, neuronal degenerative diseases, and cancers [6,7,8,9,10,11,12]. For example, many human tumors, such as non-small cell lung cancer, squamous cell carcinoma of the head and neck, glioblastoma, pancreatic cancer, ovarian cancer, breast cancer, and prostate cancer were found to closely relate to the over-activation of human epidermal growth factor receptor 2 (Her2), a sub-family of ErbB (erythroblastic oncogene B) protein tyrosine kinases [8,10,11,12,13,14,15]. Meanwhile, dysregulation of hematopoietic cell kinase (Hck), a Src family protein tyrosine kinase [16], was found to associate with many human diseases, including cancers, autoimmune diseases, and inflammation [17]. Hence, it is essential to determine the activity of tyrosine kinases to reveal the development and progression of diseases and to understand the molecular mechanisms leading to the diseases. 

To detect the activity of protein tyrosine kinases, several direct methods, i.e., ^32^P-phosphate labeling [18], fluorescence-labeling [19] and mass spectrometry analysis [20], and indirect methods, i.e., detection with a phosphoryl tyrosine-specific antibody [21] and the quantum dot-based method [22], were developed and widely used. However, these conventional methods exhibit different disadvantages. For example, safety is the major concern of using radio isotope labeling; while the detection of protein phosphorylation by indirect methods, i.e., a phosphoryl tyrosine-specific antibody and the quantum dot-based method, suffered from high reagent cost and complicated detection procedures. Although a trace phosphorylation of residues can be sensitively detected on the mass spectrometer, the expansive instrumentation blocks its extensive usage by scientists. Hence, a rapid, simple, and effective method for the detection of protein tyrosine kinase activity in vitro would be favorable for clinical diagnosis, prognosis of drug treatment, and drug design and screening.

Electrochemical methods have been developed for the detection of interactions between protein–protein [23,24,25], protein–DNA [26] and enzymatic reactions, including the protein tyrosine kinases activity [27,28,29,30,31,32,33,34]. These methods can be categorized into label-based and label-free methods, based on the sources of redox responses. The label-based methods are mainly based on the labeling of the electro-inactive phosphate group with electroactive species, such as ferrocene-conjugated ATP [28,29,30] and catalytic components, such as gold nanoparticles and redox enzyme [31,32,33,34], whereas the label-free methods are based on the direct oxidation of tyrosine residues with or without amplifications [35,36,37,38]. Although both methods exhibited high sensitivity to detect tyrosine phosphorylation, the label-free methods are more direct without additional steps of labeling. 

Recently, a novel label-free protein tyrosine kinase biosensor was reported by oxidizing the tyrosine residue(s) on the substrates of protein tyrosine kinases by tyrosinase to generate L-DOPA quinone, which was then reduced on the electrode to give a reductive response. The tyrosinase-mediated tyrosine oxidation can be blocked by the phosphorylation and suppress the reductive responses [38]. Thus, the tyrosine kinase activity can be revealed on the biosensor by the decrease in the reductive responses of their substrates. This result suggests that this biosensor can be utilized to detect the activity of various protein tyrosine kinases. In this study, a tyrosinase-based miniature protein tyrosine kinase sensing platform was developed for the quick detection of the activity of various protein tyrosine kinases, such Hck and Her2, in a small sample volume.

## 2. Materials and Methods

### 2.1. Materials

Carbon fiber paper (CFP, MGL190) was purchased from AvCarb (Lowell, MI, USA). Tyrosinase, CP-724714, Src inhibitor-1 (src-I1), HEPES, chitosan, N-hydroxysuccinimide (NHS) and N-(3-dimethylaminopropyl)-N′-ethylcarbodiimide hydrochloride (EDC) were purchased from Sigma-Aldrich (Darmstadt, Germany). Hck and Her2 were bought from SignalChem (BC, Canada). ELMO-Y511 (QNLS*Y*TEIL, a Hck peptide substrate) [39], FLT3 (DNE*Y*FYV, a Her2 peptide substrate) [29], c-Src substrate-1 (YI*Y*GSFK, a c-Src peptide substrate) [38], and kemptide (LRRASLG, a PKA peptide substrate) were synthesized by AngeneBiotech (Taipei, Taiwan). MgCl_2_ was purchased from Yakuri Pure Chemical Co. (Kyoto, Japan). Sodium dihydrogen phosphate, sodium hydrogen phosphate and MnCl_2_ were supplied by SHOWA (Kumamoto, Japan). DMEM (Dulbecco’s Modified Eagle’s medium) and fetal bovine serum were obtained from HyClone Laboratory Inc. (Marlborough, MA, USA).

### 2.2. Preparation of Tyrosinase-Based Electrode

The working area (0.3 × 0.3 cm^2^) of a CFP strip (0.3 × 1.0 cm^2^) was first cleaned with oxygen plasma using a plasma cleaner (Atto, Diener Electronic, Ebhausen, Germany). The plasma treatment was performed under the oxygen pressure of 0.4 N and a power of 75 watts for 15 s. After plasma treatment, the CFP strip was rinsed once with double deionized water (d.d. H_2_O), followed by spreading 2 μL of 0.4% chitosan/1% acetic acid solution on the working area and then air-dried. The immobilization of tyrosinase (200 U) was performed by first mixing with the mixture of 10 mM NHS and 10 mM EDC in 50 mM phosphate buffer, pH 6.6. After incubating at room temperature for 15 min, the tyrosinase/EDC/NHS mixture (6 μL) was then spread on the working area of CFP under the room temperature for at least 2 h.

### 2.3. Fabrication of the Miniature Protein Tyrosine Kinase Sensing Platform

The miniature tyrosine kinase sensing platform consists of a polylactic acid (PLA) holder, a working electrode, a platinum counter electrode and a Ag/AgCl reference electrode (Figure S1, Supplemental information). The PLA holder, generated by the 3D printer (Botfeeder Co., Taiwan), is a rectangular block (4.2 × 4.2 × 7.5 cm^3^) with a cavity of 1.8 × 1.9 × 5.7 cm^3^. It is composed of two parts: (a) the desk-like top part (~5.5 cm height) contains a hole of 0.8 cm in diameter to place the reference electrode; (b) the C-shaped bottom part (~2 cm height) contains a rectangular cleft of 0.3 × 0.05 cm^2^ to fix the working electrode. Once the holder was assembled, the counter (Pt wire) and reference electrodes (CH Instruments, West Lafayette, IN, USA) were fixed on top of the working electrode at a vertical distance of 2 mm (Figure S1).

### 2.4. Protein Tyrosine Kinase Reaction

The kinase reaction of Hck and Her2 was performed by mixing 2 μL of protein kinase stock solution with 18 μL kinase reaction mixture (60 mM HEPES, pH 7.5, containing 3 mM MgCl_2_, 3 mM MnCl_2_, 100 μM substrate peptide and 0.5 mM ATP) in a microfuge tube and incubated at 30 °C for a period of time.

### 2.5. Electrochemical Measurement

**Figure 1:**
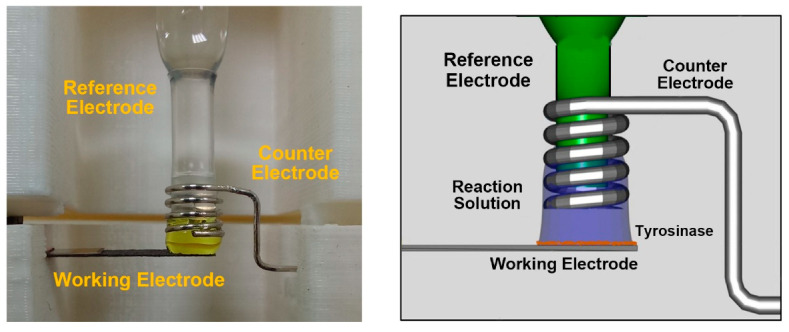
Close up view of the miniature protein tyrosine kinase sensing platform. The organization of working, counter and reference electrodes in the platform was revealed in the picture (**Left**) and the diagram (**Right**).

The electrochemical responses of the tyrosine kinases on the miniature sensing platform were determined by amperometric current–time responses (i–t curve). The space between working and counter/reference electrodes (Figure 1) was first filled with 18 μL of 100 mM phosphate buffer (pH 6.5). The electrochemical measurement was started by injecting 1–2 μL peptide stock solution of kinase reaction mixture into the phosphate buffer with pipetting and monitoring the electrochemical responses under the potential of −0.2 V vs. Ag/AgCl [38] on the electrochemical analyzer CHI 6116e (CH Instruments, West Lafayette, IN, USA). Kinase activity of Hck and Her2 was determined by the net electrochemical responses before and after phosphorylation.

## 3. Results and Discussion

### 3.1. Characterization of Miniature Detection Platform

**Figure 2:**
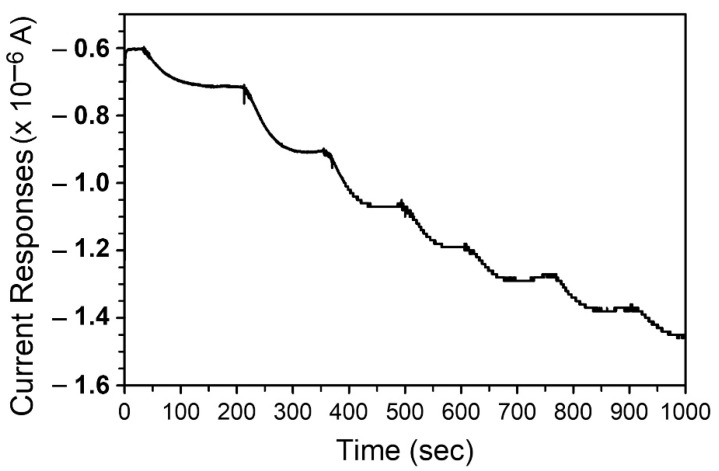
The current–time curves of c-Src substrate 1 on the miniature sensing platform. Two microliter c-Src substrate 1 stock solution (500 μM) were successively added into 100 mM phosphate buffer, pH 6.5. The initial volume of phosphate buffer is 18 μL. The i-t responses of peptide were monitored under a potential of −0.2 V vs. Ag/AgCl.

A miniature protein tyrosine kinase sensing platform (Figure 1 and Figure S1) with a three-electrode setup was developed that allowed the detection of peptide substrates in a volume as small as 18 μL (Figure 1). The ability of the miniature sensing platform to detect the peptides with tyrosine residue(s) was demonstrated by measuring the i–t responses of c-Src substrate 1 peptide, which was successively added into the phosphate buffer (Figure 2). The step current responses were observed under the potential of −0.2 V (with a response time from 85 to 42 s), suggesting the capability of the miniature sensing platform to detect the tyrosine residue-bearing peptides without regenerating the electrode surface. However, following the successive addition of c-Src substrate 1, the responses decreased gradually. It may be due to the increase in detection volume during the successive measurements that lead to dilution of the peptide concentrations. This hypothesis could be demonstrated by plotting the responses curves of c-Src substrate 1 concentrations vs. current responses from the results of Figure 2. Since the volume of electrolyte expanded from 18 μL to 32 μL upon the successive injection of c-Src substrate 1 stock solution, the final concentration of peptide after each addition could then be calculated by multiplying the corresponding dilution factor. As shown in Figure S2, the current responses of c-Src substrate 1 was linearly proportional to i-t concentration after adjustment with an R^2^ of 0.998. The developed miniature tyrosinase-based tyrosine kinase sensing platform exhibited a repeatability of 6.6% RSD (Relative standard deviation) for a repetitive measurement of 50 μM c-Src substrate I (n = 6) (Figure S4).

The specificity of the miniature sensing platform to recognize peptide with tyrosine residues was further demonstrated by alternately adding 10 μM FLT3, a Her2 peptide substrate, and 100 μM kemptide, and a PKA peptide substrate into the phosphate buffer (Figure S3A, Supplemental information). The electrochemical responses occurred only when FLT3 was added into the phosphate buffer. Similar results were also observed when ELMO-Y511, a Hck peptide substrate, and kemptide were alternately added (Figure S3B). Compared to the previously reported experimental setup [38], a smaller detection volume is needed for the developed miniature sensing platform. The electrolyte required for analysis was greatly reduced from 10 mL to 0.02 mL.

### 3.2. Detection of Her2 and Hck Activity on the Miniature Sensing Platform

**Figure 3:**
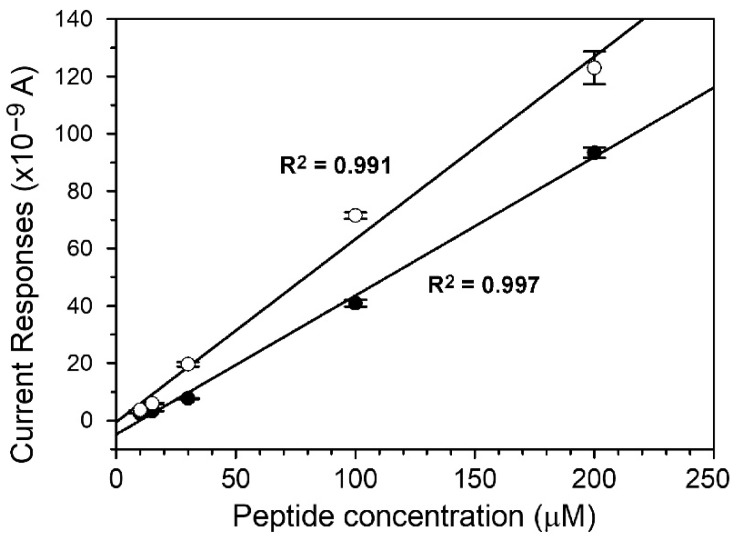
Dose responses of ELMO-Y511 and FLT3 peptides. The electrochemical responses of various concentrations (0, 10, 15, 30, 100 and 200 μM) of ELMO-Y511 (closed circle) and FLT3 (open circle) were determined on the miniature sensing platform. The data is mean ± S.D of three independent experiments.

Previously, the miniature tyrosine kinase sensing platform was shown to be able to detect peptide substrates of protein tyrosine kinase, i.e., ELMO-Y511 and FLT3. To further elucidate the capability of miniature sensing platform in detecting the tyrosine kinase activity the dose-dependent responses and time-dependent phosphorylation of ELMO-Y511 and FLT3 were investigated. As shown in Figure 3, ELMO-Y511 (closed circle) and FLT3 (open circle) could be detected in a linear range of 10 to 200 μM with the R^2^ of 0.997 and 0.991, respectively.

**Figure 4:**
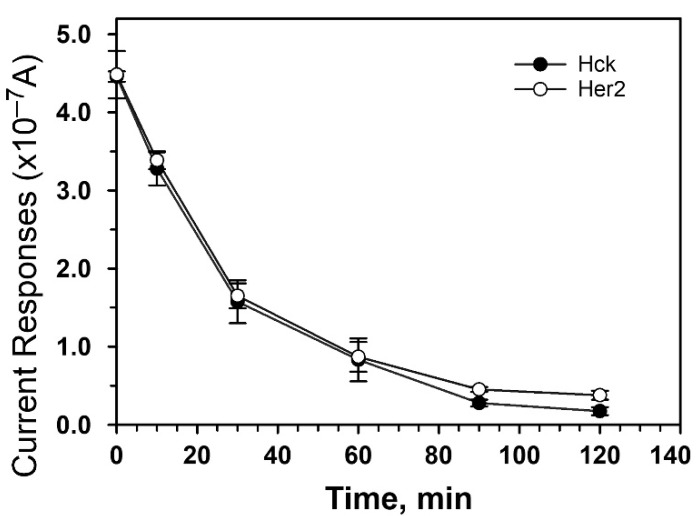
Time-dependent phosphorylation of peptide substrates by Hck and Her2. The phosphorylation of 100 μM ELMO-Y511peptide (close circle) and 100 μM FLT3 peptide (open circle) were performed by Hck (10 U/mL) and Her2 (10 U/mL), respectively, at 30 °C for 0, 10, 30, 60, 90 and 120 min. At each time point, 2-μL of reaction mixture was withdrawn and subjected to electrochemical measurement under the potential of −0.2 V. The data is mean ± S.D of three independent experiments.

A time-dependent phosphorylation of ELMO-Y511 and FLT3 peptides by Hck (10 U/mL) and Her2 (10 U/mL), respectively, was also determined at 30 °C for 0, 10, 30, 60, 90, and 120 min (Figure 4). The reductive current of ELMO-Y511 and FLT3 peptides without phosphorylation were 4.45 × 10^−7^ ± 6.70 × 10^−9^ A and 4.48 × 10^−7^ ± 3.03 × 10^−9^ A, respectively. Upon the phosphorylation with tyrosine kinases, e.g., Hck and Her2, the reductive current of ELMO-Y511 and FLT3 peptides (i.e., 3.27 × 10^−7^ ± 2.13 × 10^−8^ A and 3.38 × 10^−7^ ± 1.13 × 10^−8^ A, respectively) reduced about 25% after 10 min reaction and reduced over 50% (i.e., 1.57 × 10^−7^ ± 2.75 × 10^−8^ A and 1.65 × 10^−7^ ± 1.60 × 10^−8^ A, respectively) after 30 min reaction. After 90 min phosphorylation, the electrochemical responses of both peptides reached plateau with a reductive current of 2.78 × 10^−8^ ± 4.26 × 10^−9^ for ELMO-Y511 and 4.52 × 10^−8^ ± 3.06 × 10^−9^ for FLT3. This result suggests that the activity of Hck and Her2 can be detected on the miniature sensing platform in a kinase reaction for as short as 10 min.

**Figure 5:**
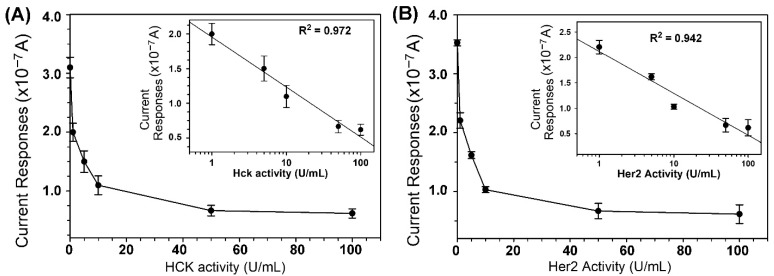
Phosphorylation of ELMO-Y511 and FLT3 peptides by various activities of Hck and Her2. The kinase reaction was performed in kinase reaction mixture containing 0.5 mM ATP,100 μM ELMO-Y511 or FLT3 peptide and various activities (0, 1, 5, 10, 50, 100 U/mL) of Hck (**A**) and Her2 (**B**). Reaction was performed at 30 °C for 30 min. Subsequently, reaction mixture (2 μL) was subjected to electrochemical measurement under the potential of −0.2 V. The inset of each panel is the semi-log plot of the same set of data. The data is mean ± S.D of three independent experiments.

The phosphorylation of ELMO-Y511 and FLT3 peptides by various activities (0, 1, 5, 10, 50, and 100 U/mL) of Hck and Her2, respectively, was carried out at 30 °C for 30 min prior to the electrochemical measurement. As shown in Figure 5, the phosphorylation of peptides increased linearly with the logarithm of the activity of Hck (Figure 5A and inset; R^2^ = 0.972) and Her2 (Figure 5B and inset; R^2^ = 0.941) with the lowest detection limit of 1 U/mL (S/N ≥ 3) to Hck and Her2. 

### 3.3. The Effect of Inhibitors to the Activity of Hck and Her2

**Figure 6:**
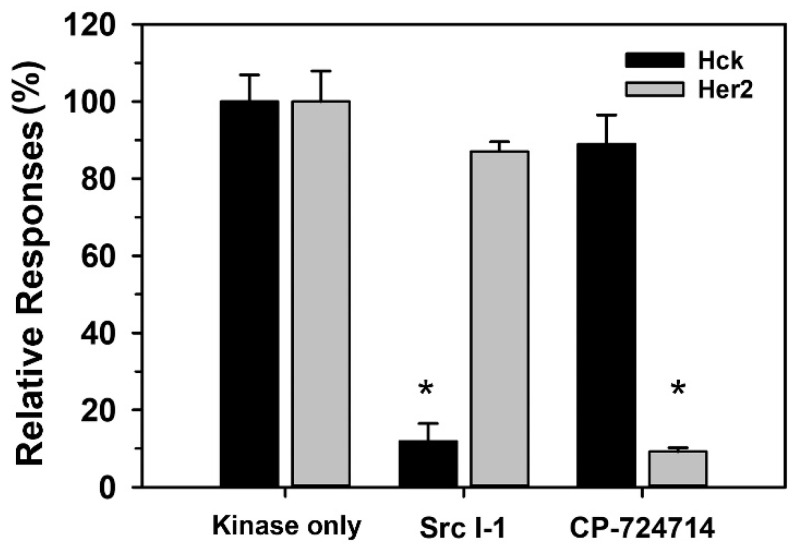
Effect of inhibitors on the activity of Hck and Her2 protein kinases. The phosphorylation reactions were performed in a kinase reaction mixture containing 10 U/mL of Hck (closed bar) or Her2 (open bar) and 100 μM ELMO-Y511 (for Hck) or FLT3 (for Her2) with or without 176 nM Src-I1 or 20 nM CP-724714. After reaction, 2 μL reaction mixture were subjected to electrochemical measurement on the sensing platform. The relative responses upon phosphorylation in the presence and absence of inhibitors were calculated and presented as the mean ± S.D of three independent experiments. (* denote significantly different from the activity of “kinase only” at *p* < 0.05).

Protein tyrosine kinase inhibitors are widely used in the laboratory to elucidate the signaling pathway as well as in the clinic to treat cancers. The effect of inhibitors can be accessed by the decrease in the protein tyrosine kinase activity after treatment. Therefore, the capability of the developed miniature tyrosine kinase sensing platform in studying the inhibitory effect of Src-I1, the inhibitor of Src family kinases [40], and CP724714, the Her2 specific inhibitor [41], on the corresponding kinases was studied. As shown in Figure 6, the activity of Hck, a Src family kinase, could be suppressed about 88% in the presence of 176 nM Src I-1, whereas only about 13% of Her2 activity was inhibited. In contrast, 20 nM CP-72471 could inhibit Her2 activity by about 91%, but only slightly affected the Hck activity by around 12%. 

### 3.4. Interference Effect of Cultural Medium to the Protein Tyrosine Kinase Activity

**Figure 7:**
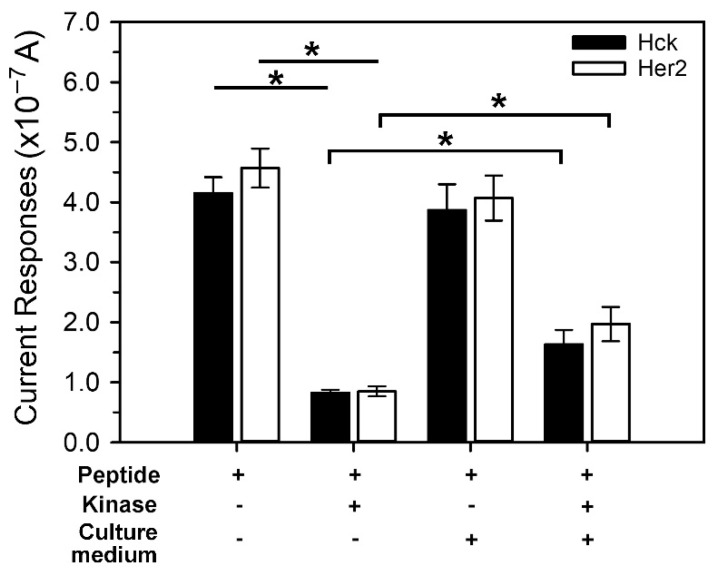
Interference effect of culture medium on the detection of peptides of protein tyrosine kinases. Peptide stock solution and protein kinase stock solution were diluted with the cell culture medium in a ratio of 1:1. The phosphorylation was performed in a kinase reaction mixture containing 10 U/mL of Hck (closed bar) or Her2 (open bar) and 100 μM ELMO-Y511 (for Hck) or FLT3 (for Her2). The data is mean ± S.D of three independent experiments. (* *p* < 0.05).

Generally, most of the protein tyrosine kinases are either membrane bound or residing in the cytoplasm. Hence, it is usually required to prepare samples from cellular extract for protein kinase assay. To understand the effect of the cell remnants or intracellular constituents in causing the interference with the electrochemical measurement, the culture medium (DMEM containing 10% fetal bovine serum) was used to prepare the kinase reaction mixture that contains peptide substrate, i.e., ELMO-Y511 or FLT3, and protein tyrosine kinases, i.e., Hck or Her2. The reductive current of ELMO-Y511 and FLT3 alone was 4.15 × 10^−7^ ± 2.66 × 10^−8^ A (closed bar) and 4.57 × 10^−7^ ± 3.25 × 10^−8^ A (open bar), respectively (Figure 7, peptide only). When mixed with the culture medium, the electrochemical responses of ELMO-Y511 and FLT3 (peptide + culture medium) decreased slightly to 3.87 × 10^−7^ ± 4.34 × 10^−8^ A and 4.07 × 10^−7^ ± 3.78 × 10^−8^ A, respectively. This result suggests that a cell culture medium or even a cell crude extract may not affect the electrochemical measurement of peptides on the sensing platform.

In contrast, the activity of Hck and Her2 was moderately affected by the culture medium. This is demonstrated by the finding that the phosphorylated form of ELMO-Y511 (dark bar) and FLT3 (bright bar) decreased in the kinase reaction mixture containing the culture medium (Figure 7). The ELMO-Y511 and FLT3 peptides phosphorylating without the culture medium exhibited a response (peptide + kinase) of 8.26 × 10^−8^ ± 5.31 × 10^−9^ A and 8.51 × 10^−8^ ± 8.39 × 10^−9^ A, respectively; while in the presence of culture medium, the responses of peptides (kinase + culture medium) changed to 1.63 × 10^−7^ ± 2.44 × 10^−8^ A and 1.97 × 10^−7^ ± 2.88 × 10^−8^ A, respectively. This result indicates that the kinase activity of Hck and Her2 was suppressed 33% and 43%, respectively, by the culture medium. Although the exact mechanism underlying the medium-mediated inhibition of kinase activity is not clear, the presence of kinase inhibitors, phosphatases, proteases and/or thiol compounds, in the culture medium is postulated. Phosphotyrosine phosphatases are known to remove the phosphate group from the phosphotyrosines; while proteases contaminants can degrade protein kinases. The contamination of both substances may result in the underestimation of kinase activity. Thiol compounds, such as cysteine and glutathione, could block the reaction of tyrosinase by forming the inactive conjugates from the intermediates [42]. To avoid the influence of cellular components on the kinase reaction, the reaction mixture for protein tyrosine kinase reactions is suggested to be reformulated, such as adding phosphatase inhibitors and sample pretreatment.

## 4. Conclusions

In this work, a simple and label-free miniature sensing platform for detecting the activity of protein tyrosine kinases was developed and characterized. Compared to the previous experimental setup [38] and published reports (Table 1), the newly developed platform is simple in design, easy to operate, and requires only 1–2 μL samples for kinase activity assay. With the reduction of detection volume, the sample volume required for analysis was also significantly decreased from 20 μL to 2 μL. Simple in design and easy operation are also the advantages of the current experimental setup. These studies showed that the current experimental setup exhibits great potential for the detection of biological samples that are usually rare and expensive to acquire in large quantity. The unique tyrosinase-based detection mechanism [38] allows the developed miniature sensing platform to detect different protein tyrosine kinases on the same electrodes. This is demonstrated by the observation that both Hck and Her2 activity can be successively detected without changing electrodes. The platform was also effective in assessing the specificity of inhibitors on different tyrosine kinases, indicating that the platform can be used to monitor and screen the effect of drugs on different tyrosine kinases in a short time. In summary, the developed tyrosine kinase sensing platform exhibits a great potential to be a powerful tool for the detection of protein tyrosine kinase activity, the screening of tyrosine kinase-based drugs, and clinical diagnoses.

## Figures and Tables

**Table 1:** Comparison of the performance of various tyrosine kinase biosensors.

Electrode Types	Working Mechanism	Reusability (R.S.D.)	Sample Volume Required (μL)	Protein Tyrosine Kinases	Linear Range of Detection	Limitation of Detection	Ref.
Miniature Tyrosinase/CFP	Tyrosinase-based Tyr Oxidation	High (6.6%)		Src	N.D.	N.D.	
			1–2 μL	Hck	1–100 U/mL	1 U/mL	This study
				HerB	1–100 U/mL	1 U/mL	
Peptide-immobilized SPCE	AuNP-based redox response	Low (N.A.)	25 μL	Src	N.A.	5 U/mL	[32]
MWCNT -modified SPCE	Direct oxidation of Tyr	High (N.A.)	20 μL	Src	N.A.	5 U/mL	[35]
Graphene-modified glassy carbon electrode	Graphene-assisted direct oxidation of Tyr	High (N.A.)	20 μL	Src	0.26 to 33.79 nM	0.087 nM	[37]
Tyrosinase/CFP	Tyrosinase-based Tyr Oxidation	High (2.87%)	20 μL	Src	1.9–237.6 U/mL	0.23 U/mL	[38]
Peptide-immobilized Gold electrode	4-mercaptophenylboronic acid (MPBA)MPBA-assisted AgNP aggregates-based redox response	Low (N.A.)	--	Src	10–80 ng/mL	1.2 ng/mL	[43]
Peptide-immobilized ITO electrode	Os(bpy)_3_^+2^-mediate Tyr oxidation	Low (N.A.)	--	EGFR	N.A.	1 U/mL	[44]

N.D., Not determined; N.A., Not applicable; MPBA, 4-mercaptophenylboronic acid; SPCE, screen-printed carbon electrode.

## Data Availability

Not applicable.

## Supplementary Materials

The following are available online at https://www.mdpi.com/article/10.3390/bios11070240/s1, Figure S1: The structure of the miniature protein tyrosine kinase sensing platform., Figure S2: The response curve of c-Src substrate 1 concentration vs. current responses; Figure S3. The chronoamperometric measurement of substrate peptides for Hck and Her2; Figure S4. Reproducibility of tyrosine kinase sensing platform.
