# Supplementary material for: Development of the Sensing Platform for Protein Tyrosine Kinase Activity
Source: Biosensors (Basel). 2021 Jul 15;11(7):240. doi: 10.3390/bios11070240 (PMC8301957; doi:10.3390/bios11070240)
Supplement: Supplementary file 1 [file biosensors-11-00240-s001.zip › biosensors-1247783-supplementary.pdf]

biosensors

MDPI

Supplemental Information

Development of the Sensing Platform for Protein Tyrosine Kinase Activity

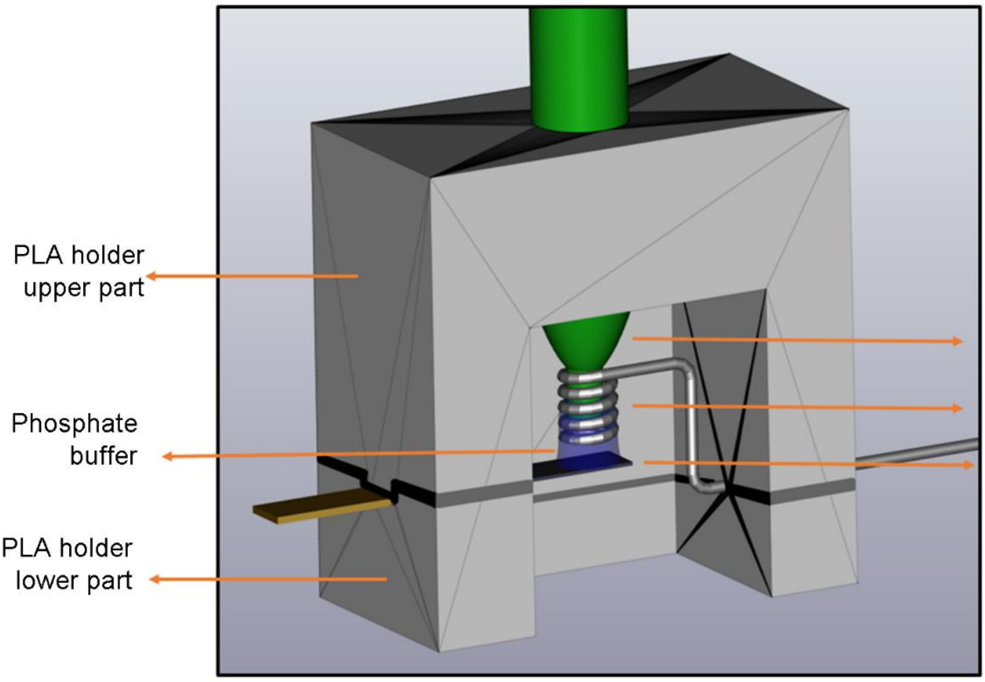

Figure S1. The structure of the miniature protein tyrosine kinase sensing platform. In this platform the working, counter and reference electrodes were fixed by a PLA holder. The PLA frame was generated by the 3D printer (Botfeeder Co., Taiwan) and composed of upper and lower parts. The PLA holder is a rectangular block (4.2 × 4.2 × 7.5 cm^3) with a cavity of 1.8 × 1.9 × 5.7 cm^3. The C-shaped bottom part (~2 cm height) contains a rectangular cleft of 0.3 × 0.05 cm^2 on the left hand Scheme 5. 5 cm height) contains a hole of 0.8 cm in diameter on the desk top to hold a Ag/AgCl reference electrode. When the holder is assembled, the counter (Pt wire) and reference electrodes (CH Instruments, West Lafayette, IN, USA) will be fixed on top of the working electrode at a vertical distance of 2 mm.

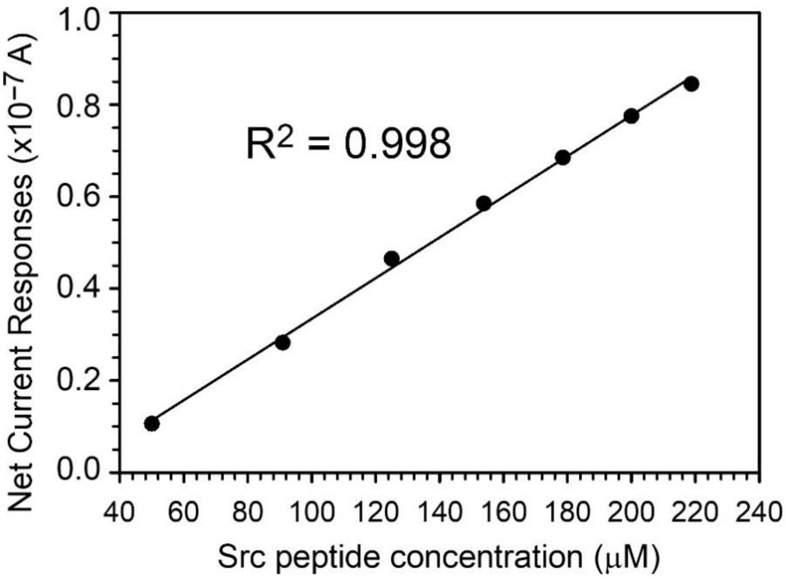

Figure S2. The response curve of c-Src substrate 1 concentration vs. current responses. The data used in this plot was derived from the results of Figure 2. The final concentrations of c-Src substrate 1 in electrolyte after each injection was calculated by Equation 1:

$$C_f = \frac{V_i\, C_i + V_s\, C_s}{V_i + V_s} \tag{1}$$

Where, C_i and C_f are initial and final concentrations of c-Src substrate 1 in the electrolyte; C_s is 500 μM that is the concentration of c-Src substrate 1 stock solution. The V_i is the volume of electrolyte before the injection of c-Src substrate 1 stock solution at each point; whereas, Vs is the volume (2 μL) of c-Src substrate 1 stock solution injected into the electrolyte each time.

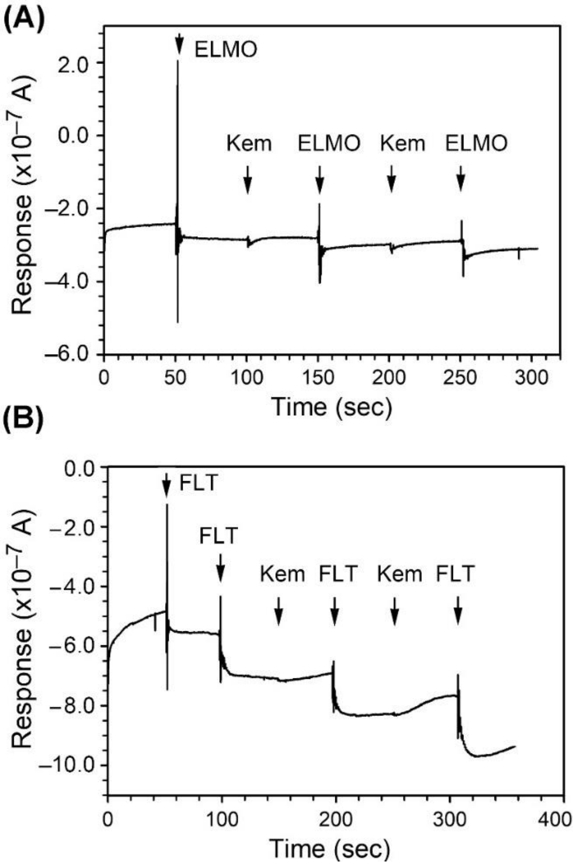

Figure S3. The chronoamperometric measurement of substrate peptides for Hck and Her2. Two tyrosine residues-bearing peptides, ELMO-Y511 (ELMO) and FLT3 (FLT) peptides, and kemptide, a peptide for a serine/threonine kinase PKA (Kem) were investigated in this study. (**A**) The ELMO-Y511 peptide (10 μM) and the kemptide (100 μM) were alternately injected into 18 μL phosphate buffer (pH 6.8). The electrochemical responses were monitored under −0.2 V. (**B**) The FLT3 peptide (10 μM) and the kemptide (100 μM) were alternately injected into 18 μL phosphate buffer (pH 6.8). The electrochemical responses were monitored under −0.2 V. The reductive currents were observed when FLT3 peptide (FLT in panel A) and ELMO-Y511 peptide (ELMO in panel B) were added into the phosphate buffer with a potential of −0.2 V. No reductive current was observed when kemptide (Kem) was added.

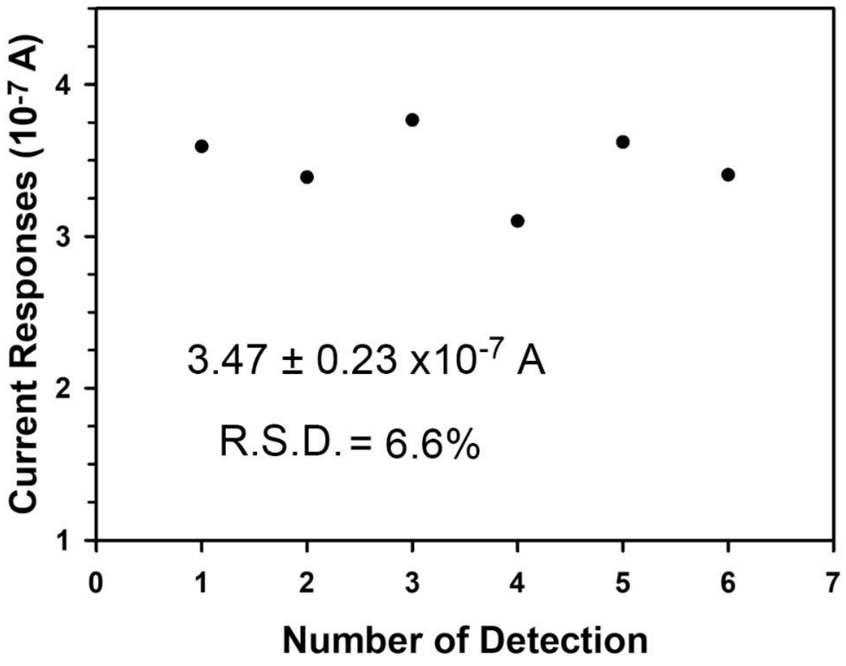

Figure S4. Reproducibility of tyrosine kinase sensing platform. The current responses of 5 μM c-Src peptide was repetitively measured on the tyrosine kinase sensing platform. After 6 measurements, the average electrochemical response was 3.47 ± 0.23 × 10^{-7} A with a R.S.D. of 6.6 %.
